# Supplementary figures and images for: Impact of dataset size and long-term ECoG-based BCI usage on deep learning decoders performance
Source: Front Hum Neurosci. 2023 Mar 16;17:1111645. doi: 10.3389/fnhum.2023.1111645 (PMC10061076; doi:10.3389/fnhum.2023.1111645)

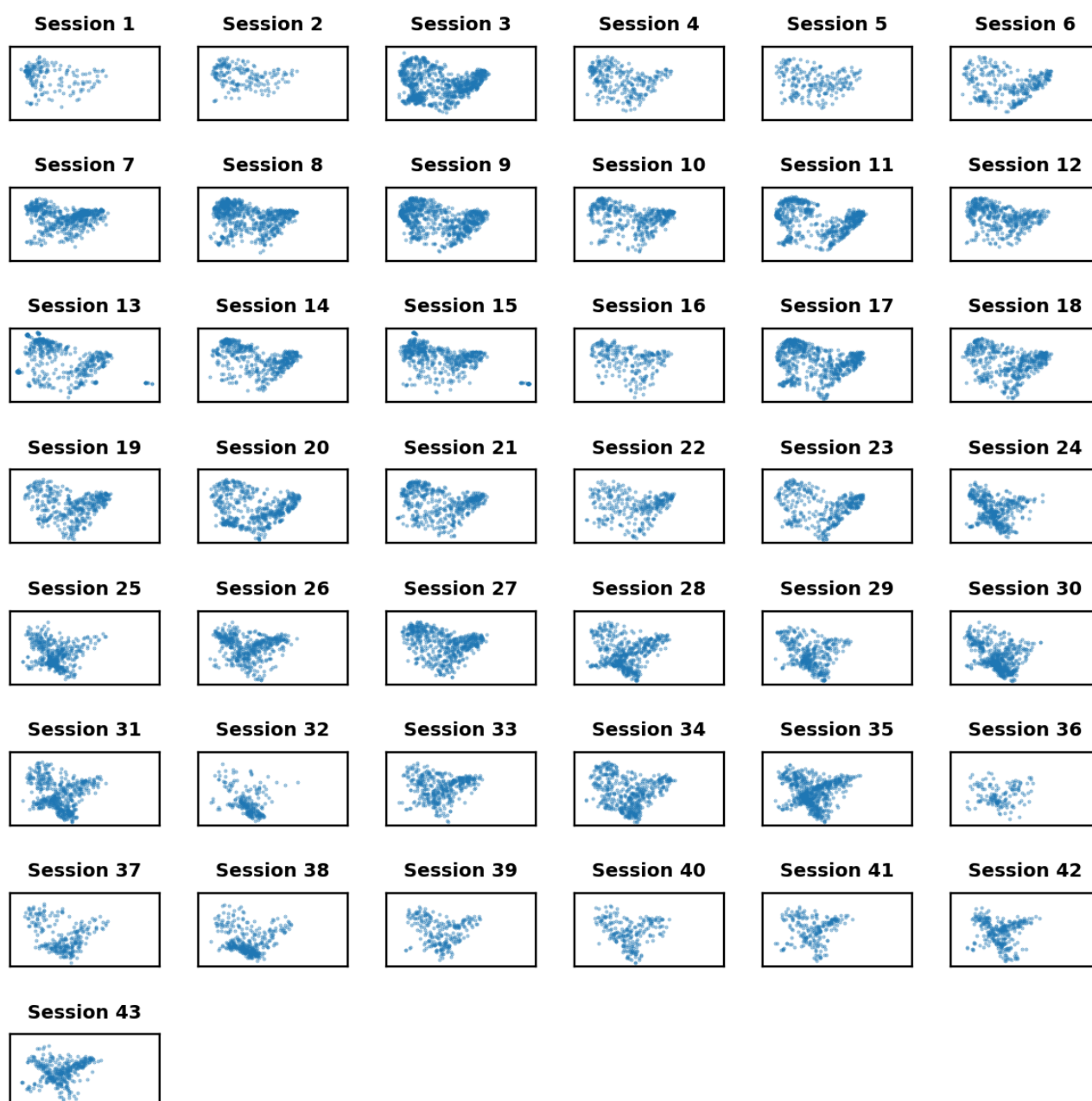

**Figure 1.** Left hand dataset embedding showed for every session separately.

Supplement: Supplementary file 1 [file Image_1.pdf]

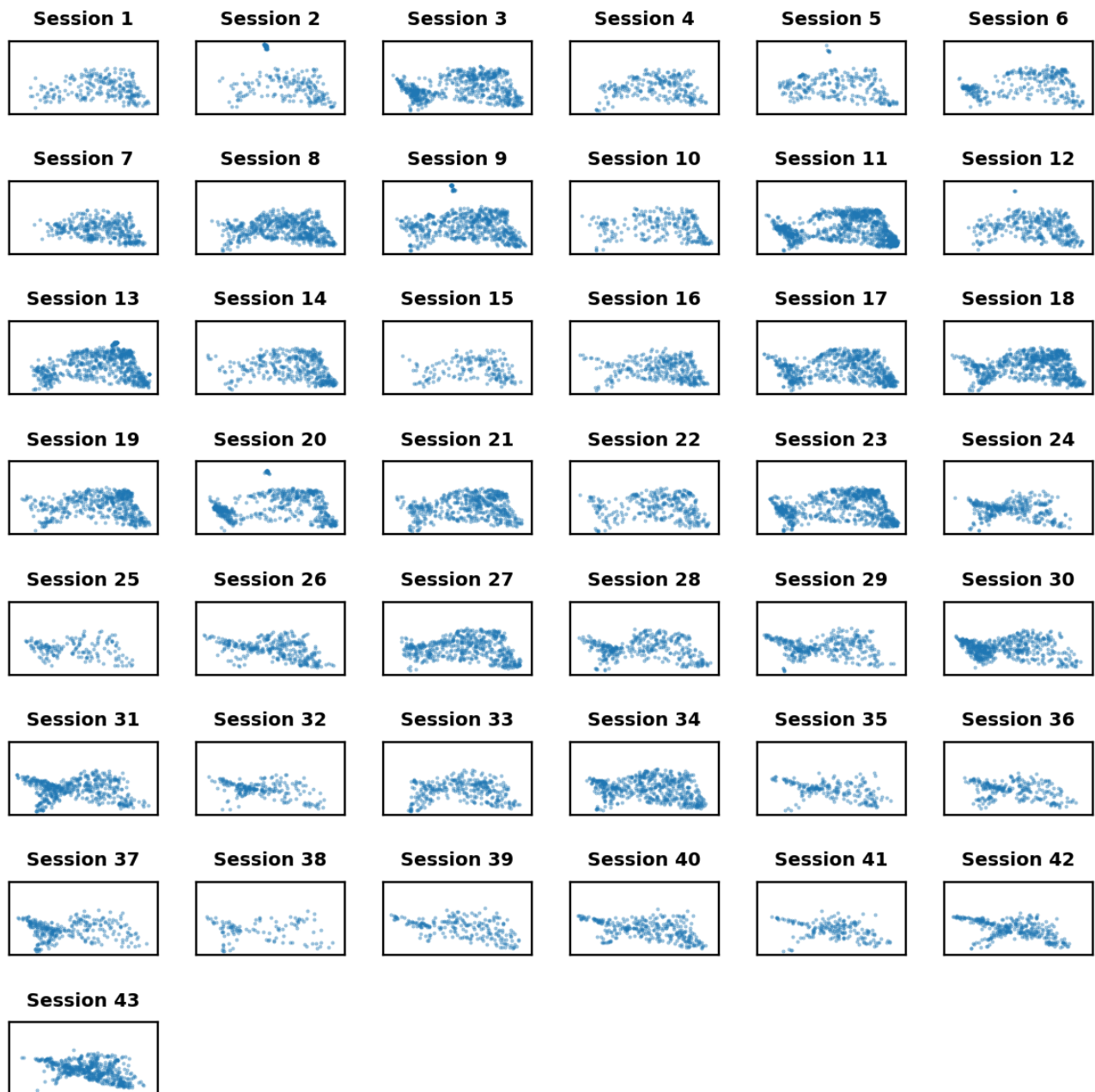

**Figure 2.** Right hand dataset embedding showed for every session separately.

Supplement: Supplementary file 2 [file Image_2.pdf]
