# Supplementary material for: Impact of dataset size and long-term ECoG-based BCI usage on deep learning decoders performance
Source: Front Hum Neurosci. 2023 Mar 16;17:1111645. doi: 10.3389/fnhum.2023.1111645 (PMC10061076; doi:10.3389/fnhum.2023.1111645)
Supplement: Supplementary file 3 [file Image_3.pdf]

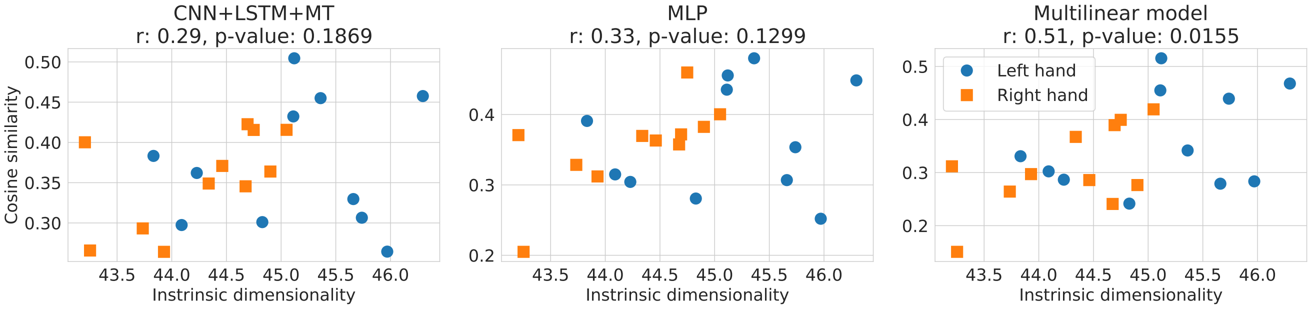

Figure 3. Relationship between cosine similarity and ID of the training dataset computed with TwoNN for dataset translation experiment. In the plot titles, Pearson correlation coefficient r and p-value (the probability of two uncorrelated inputs obtaining r at least as extreme as obtained in this case) are presented.
